# Appropriate Screening for Leishmaniasis before Immunosuppressive Treatments

**DOI:** 10.3201/eid1510.090881

**Published:** 2009-10

**Authors:** Antonio Cascio, Chiara Iaria

**Affiliations:** University of Messina, Messina, Italy (A. Cascio); Sapienza University of Rome, Rome, Italy (C. Iaria)

**Keywords:** Leishmaniasis, immunosuppression, parasites, screening, letter

## To the Editor

We read with great interest the article by Xynos et al. reporting 2 cases of leishmaniasis in patients treated with biologic drugs (*1*). Although we agree with most of the article, we are not totally convinced that serologic analysis alone could be used to screen for leishmaniasis before initiation of biologic or immunosuppressive treatments. Evidence indicates that serologic analysis can identify only symptomatic or asymptomatic cases with recent and still active infection (*2*,*3*).

*Leishmania* spp. are pathogens that infect hematopoietic cells, where they establish chronic intracellular parasitism and survive for an infected person’s lifetime. In leishmaniasis-endemic countries, asymptomatic visceral leishmaniasis (VL) infections occur more frequently than clinically apparent VL cases. An estimated 10%–20% of persons with asymptomatic infections develop clinically overt VL (*4*). The leishmanin skin test (LST) (Montenegro test), an intradermal injection of a suspension of killed promastigotes, measures delayed hypersensitivity reactions and appears to be the only screening test capable of detecting asymptomatic leishmania infections.

A positive LST result is thought to indicate durable cell-mediated immunity after asymptomatic infection or clinical cure of VL. LST positivity may appear months to years postinfection, but once positivity appears, it persists in immunocompetent patients. A survey of VL in Ethiopia showed LST positivity in 32.2% of the population, but leishmania antibodies were found in only 4.1% (*5*).

Because different antigen preparations may affect test sensitivity, LST should use promastigotes of the *Leishmania* spp. present in an area. We believe that ideal screening for leishmaniasis should include LST along with serologic analysis. Unfortunately, little data exist on the use of antileishmania therapies for LST-positive or serologically positive patients. VL with unusual signs and symptoms may develop in immunocompromised patients with previous LST positivity after immunosuppressive treatments. Information about LST positivity is useful for calling attention to this potential risk for VL that may have unusual manifestations in these persons.
