# Supplementary material for: Appropriate Screening for Leishmaniasis before Immunosuppressive Treatments
Source: Emerg Infect Dis. 2009 Oct;15(10):1706–7. doi: 10.3201/eid1510.090881 (PMC2829195; doi:10.3201/eid1510.090881)
Supplement: Related texts

# Letter: Appropriate Screening for Leishmaniasis before Immunosuppressive Treatments

**DOI:** 10.3201/eid1510.091024

**Published:** 2009-10

**Authors:** Ioannis D. Xynos, Maria G. Tektonidou, Dimitrios Pikazis, Nikolaos V. Sipsas

**Affiliations:** National and Kapodistrian University of Athens, Athens, Greece (I.D. Xynos, D. Pikazis, N.V. Sipsas); Euroclinic Hospital of Athens, Athens (M.G. Tektonidou).

## In Response

In their letter responding to our recently published article (*1*) Cascio and Iaria spark an important discussion on the usefulness of screening for *Leishmania* infection before administering biologic agents or other immunosuppressive treatments to patients having autoimmune rheumatic diseases and living in areas where *Leishmania* parasites are endemic (*2*). Although we agree in principle that early detection of asymptomatic *Leishmania* infection will decrease the incidence of the disease in immunosuppressed patients, current diagnostic tools may have a limited (or restricted) role in detecting *Leishmania* infection in this vulnerable patient population. Screening for leishmaniasis has been hampered by the lack of a standard test. Currently available serologic methods have variable sensitivities, specificities, and cross-reactivities, depending on the species being tested and the region where tests are performed. Many experts believe that serologic tests may complement other existing diagnostic tools, raising cost-efficiency concerns, especially in financially deprived countries (*3*).

A positive leishmanin skin test (LST) result indicates exposure to *Leishmania* spp. and is generally thought to reflect a durable cell-mediated immune response. No cross-reaction occurs in patients with Chagas disease, but some cross-reactions are found in patients with glandular tuberculosis or lepromatous leprosy (*4*). Sustained positive responses have been documented for up to 20 years after exposure to the *Leishmania* parasite. Nevertheless, LST has limitations. In a longitudinal study of visceral leishmaniasis in Bangladesh, Bern et al. reported loss of LST sensitivity attributed to antigen-production issues, such as standardization and documentation of sensitivity, potency, and stability of leishmanin antigens (*5*). Also, prior treatment with immunosuppressive agents, which influence cell-mediated immunity, may decrease LST prognostic potency similarly to changes observed for the tuberculin skin test in similar settings (*6*).

Variations in specificities and sensitivities limit the diagnostic potential of available diagnostic tools. The context of immunosuppression further contributes to the diagnostic complications and increases the need for additional research in leishmaniasis diagnostics.

## References

[1] Xynos ID, Tektonidou MG, Pikazis D, Sipsas NV. Leishmaniasis, autoimmune rheumatic disease, and anti–tumor necrosis factor therapy, Europe. Emerg Infect Dis. 2009;15:956–9. 10.3201/eid1506.09010119523302PMC2727307

[2] Cascio A, Iaria C. Appropriate screening for leishmaniasis before immunosuppressive treatments. Emerg Infect Dis. 2009;15:1705–6.10.3201/eid1510.090881PMC282919519861088

[3] Zijlstra EE, Nur Y, Desjeux P, Khalil EA, El-Hassan AM, Groen J. Diagnosing visceral leishmaniasis with the recombinant K39 strip test: experience from the Sudan. Trop Med Int Health. 2001;6:108–13. 10.1046/j.1365-3156.2001.00680.x11251906

[4] Singh S, Sivakumar R. Recent advances in the diagnosis of leishmaniasis. J Postgrad Med. 2003;49:55–60. 10.4103/0022-3859.92712865572

[5] Bern C, Amann J, Haque R, Chowdhury R, Ali M, Kurkjian KM, Loss of leishmanin skin test antigen sensitivity and potency in a longitudinal study of visceral leishmaniasis in Bangladesh. Am J Trop Med Hyg. 2006;75:744–8.17038705

[6] Lalvani A, Millington KA. Screening for tuberculosis infection prior to initiation of anti-TNF therapy. Autoimmun Rev. 2008;8:147–52. 10.1016/j.autrev.2008.07.01118706526PMC2593775
